# New Drugs, Appliances, and Things Medical

**Published:** 1890-01-11

**Authors:** 


### NEW DRUGS, APPLIANCES AND THINGS MEDICAL.

[All preparations, appliances, novelties, etc., of which a notice is desired, should be sent to The Editor, The Lodge, Porchester Square, W.]

### GEORGE MASON AND CO.'S CONCENTRATED MEAT PREPARATIONS AND O.K. SAUCE.

The meat preparations are various forms of concentrated beef-tea and bouillon. We have tested these, and find them what they profess to be. They have good nutritive value, and the O.K. Bouillon especially has a very considerable quantity of albuminous constituents. The firm also puts up some very neat boxes of concentrated meat lozenges. Of these we can speak personally as a useful preparation, containing a good deal of stimulating nourishment in a condensed and convenient form. But after all is said, the main test of these invalid foods is summed up in the questions, does the patient like them? and does he do well on them? With regard to Messrs. Mason and Co.'s preparations, we can state patients do like them, and receive benefit from them, nor is the cause of this far to seek. It is simply that the nutritious beef-tea and soups, made with this firm's productions, are palatable. The ordinary form of meat extract has no food value; it is simply a mass of salts and extractives, and, as such, acts as a stimulant only. As usually met with, it is somewhat repellant, having too much the flavour and appearance of burnt meat gravy. The more enterprising of invalid food purveyors have recognised this drawback, and made their preparations more sightly, tasty, and nutritious. The O.K. Sauce, which the above firm produces, is a very pleasant adjunct to those dishes requiring the aid of a digestive excitant. We notice that many of the largest London and provincial hospitals use Mason's preparations. This is sufficient evidence of the worth of the well-known "Sister of Mercy" brand.

